# Treatment With Diflunisal in Domino Liver Transplant Recipients With Acquired Amyloid Neuropathy

**DOI:** 10.3389/ti.2022.10454

**Published:** 2022-04-13

**Authors:** Velina Nedkova-Hristova, Carmen Baliellas, José González-Costello, Laura Lladó, Emma González-Vilatarsana, Valentina Vélez-Santamaría, Carlos Casasnovas

**Affiliations:** ^1^ Neuromuscular Unit, Neurology Department, Bellvitge University Hospital-IDIBELL, Barcelona, Spain; ^2^ Multidisciplinary Unit of Familial Amyloidosis, Bellvitge University Hospital-IDIBELL, Barcelona, Spain; ^3^ Liver Transplantation Unit, Bellvitge University Hospital-IDIBELL, Barcelona, Spain; ^4^ Advanced Heart Failure and Transplantation Unit, Cardiology Department, Bellvitge University Hospital-IDIBELL, Barcelona, Spain; ^5^ Neurometabolic Diseases Group, Bellvitge Biomedical Research Institute (IDIBELL), Barcelona, Spain; ^6^ Biomedical Research Network Center in Rare Diseases (CIBERER), Valencia, Spain

**Keywords:** diflunisal, transthyretin, amyloidosis, domino liver transplant, neuropathy

## Abstract

**Objectives:** To analyze the efficacy and tolerability of diflunisal for the treatment of acquired amyloid neuropathy in domino liver transplant recipients.

**Methods:** We performed a retrospective longitudinal study of prospectively collected data for all domino liver transplant recipients with acquired amyloid neuropathy who received diflunisal at our hospital. Neurological deterioration was defined as an score increase of ≥2 points from baseline on the Neurological Impairment Scale/Neurological Impairment Scale-Lower Limbs.

**Results:** Twelve patients who had received compassionate use treatment with diflunisal were identified, of whom seven had follow-up data for ≥12 months. Five patients (71.4%) presented with neurological deterioration on the Neurological Impairment Scale after 12 months (*p* = 0.0382). The main adverse effects were cardiovascular and renal, leading to diflunisal being stopped in five patients and the dose being reduced in two patients.

**Conclusion:** Our study suggests that most domino liver transplant recipients with acquired amyloid neuropathy will develop neurological deterioration by 12 months of treatment with diflunisal. This therapy was also associated with a high incidence of adverse effects and low treatment retention. The low efficacy and low tolerability of diflunisal treatment encourage the search for new therapeutic options.

## Graphical Abstract

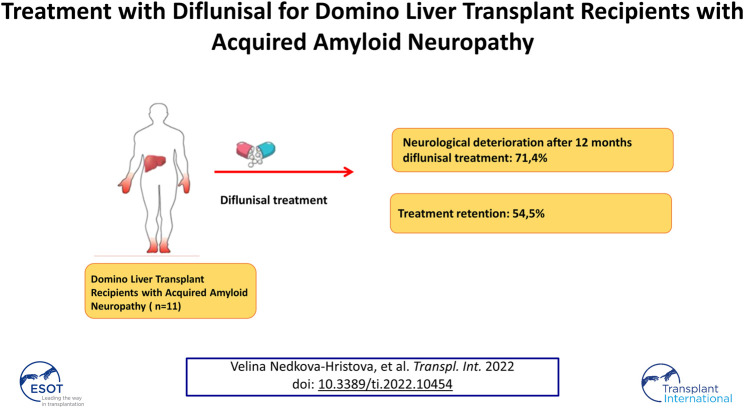

## Introduction

Hereditary transthyretin amyloidosis (hATTR) is an autosomal dominant hereditary disease caused by a mutation in the transthyretin gene, which codes for the protein of the same name [1]. Transthyretin (TTR) is dissociated into dimers and monomers that precipitate to form amyloid aggregates that are deposited in various organs [2]. One of the main manifestations is length-dependent axonal polyneuropathy that initially affects small fibers and causes painful dysesthesias and numbness [3].

Given that TTR production mainly occurs in the liver, orthotopic liver transplant has been the main treatment strategy for years. Recently, nonsurgical options have emerged to treat familial amyloid polyneuropathy (FAP), including stabilizer therapies (tafamidis and diflunisal) and transthyretin silencers (inotersen and patisiran) [4]. Diflunisal is a nonsteroidal anti-inflammatory drug and a nonspecific tetramer stabilizer that has been used off‐label to treat hATTR. Tafamidis, which binds to the unoccupied thyroxine binding sites of tetrameric TTR and prevents its dissociation into monomers [5, 4], has been approved in Europe for the treatment of hATTR amyloidosis in adults with early-stage symptomatic polyneuropathy [4]. Inotersen and patisiran reduce TTR protein by degrading nuclear TTR messenger RNA (inotersen) and forming a cytoplasmic RNA‐induced silencing complex (patisiran) [4–8]. Patisiran and inotersen have received authorization for the treatment of neuropathy in patients with both early and late disease [4].

When orthotopic liver transplant is performed, the removed liver is functionally healthy and can be donated to another patient with liver failure in domino liver transplantation (DLT) [9–12, 13, 14–20]. The graft gradually produces mutated TTR in the recipient, and over time, this can result in iatrogenic acquired amyloid neuropathy (AAN). As of December 2017, there had been 1,234 DLTs worldwide from donors with FAP [21]. However, the first cases of AAN began to be reported in these patients from 2005 [11, 12, 22, 23]. When DLT recipients develop neuropathies, few treatment options exist. Liver re-transplantation, which can stabilize or even improve symptoms [11, 12, 24], may be considered but is often limited by the patient’s age or comorbidities. Regarding medical treatment, case reports have suggested that treatment with TTR stabilizers (diflunisal or tafamidis) can produce clinical stabilization in some cases [25–27]. To date, there have been no data from case series with long-term follow-up of the effects of diflunisal or other treatments in these patients.

In this report, we aimed to describe our experience in our tertiary care center of the efficacy and tolerability of diflunisal for neurological symptoms in DLT recipients with AAN.

## Materials and Methods

### Study Design and Variables

In this retrospective longitudinal study, data were collected from the electronic medical records of patients who developed AAN after DLT and treated with compassionate-use diflunisal between 2014 and 2019 at our hospital. All DLT recipients underwent prospective routine annual neurological evaluations for early AAN diagnosis in the Familial Amyloidosis Multidisciplinary Unit (UMAF). Patients without medical contraindications (e.g., severe renal failure, uncontrolled cardiac failure, or arterial hypertension) started on treatment with diflunisal. We collected data from serial neurological assessment at baseline (before starting treatment), at 6 months of treatment, and annually thereafter (12 ± 2, 24 ± 2, and 36 ± 2 months). Assessment involved full neurological examination, with patients given Neurological Impairment Scale (NIS) and Neurological Impairment Scale-Lower Limbs (NIS-LL) scores and undergoing sensory and motor neurography. Neurological deterioration was defined as an increase in the NIS or NIS-LL of ≥2 points from baseline.

We also collected data on diflunisal side effects, focusing on new onset or worsening hypertension (need to start or adjust antihypertensives) or worsening of renal function (reduction in the estimated glomerular filtration rate of >10 ml/min from baseline on two consecutive measurements, or any value < 30 ml/min during treatment). Cardiac assessments were based on the New York Heart Association (NYHA) Functional Classification scale, NT-proBNP levels, echocardiography, and 99 mTc-DPD scintigraphy at baseline and during follow-up. Finally, we recorded any dose changes or therapy cessation.

Ethical approval was obtained by Ethics Committee for Drug Research of our center (reference number: EPA015/20; CCP-DIF-2020-01). The Ethics Committee for Drug Research of our center waived the need for written informed consent. We obtained verbal consent for data collection and noted this in patients’ electronic medical records. All methods were performed in accordance with the relevant guidelines and regulations.

### Statistical Analysis

Changes in NIS and NIS-LL scores were analyzed by two-tailed Student t-tests for paired data, after confirming distribution normality. *p*-values of <0.05 were considered statistically significant.

## Results

### Demographic and Clinical Data

We identified 12 DLT recipients who developed AAN in whom treatment with diflunisal was started as a compassionate use stabilising treatment, at a dose of 250 mg twice daily.

Six patients (50%) were diabetic, but all of them had excellent glycemic control (Tables 1, 2). Those with a history of alcohol use had been abstinent from alcohol before transplantation and at all follow-ups. Most recipients were graded as in NYHA class I (83.3%) two (16.7%) were class II, and none had evidence of amyloid deposits on cardiac scintigraphy with 99 mTc-DPD before receiving diflunisal. One patient developed heart failure, whereas all others remained stable, and none developed amyloid deposits on follow-up cardiac scintigraphy (Supplementary material).

**TABLE 1:** Demographic and clinical data for domino liver transplant recipients who developed acquired amyloid neuropathy.

Demographic and Clinical Data (*n* = 12)	
Gender (male), No. (%)	8 (66.6)
Personal history	
- arterial hypertension No. (%)	11 (91.6)
- Dyslipidemia No. (%)	9 (75)
- Diabetes mellitus No. (%)	6 (50)
- Insulin-dependent diabetes. No. (%)	5 (41.6)
Initial transplant indication	
- HCV LC, No. (%)	6 (50)
- HBV LC, No. (%)	2 (16.7)
- Alcoholic LC, No. (%)	1 (8.3)
- HBV and alcoholic LC, No. (%)	1 (8.3)
- HCV and alcoholic LC, No. (%)	1 (8.3)
- Autoimmune hepatitis LC, No. (%)	1 (8.3)
Age at the time of receiving DLT, mean (rang), years	57.7 (52, 65)
Age at onset of neurological symptoms, mean (rang), years	66.7 (57; 76)
Time between transplant and onset of symptoms, mean (rang), years	8.5 (5; 15)

DLT, domino liver transplant; HBV, hepatitis B virus; HCV, hepatitis C virus; LC, liver cirrhosis.

**TABLE 2:** Demographic and clinical characteristics, plus neurological changes.

	DLT Indication	Time DLT—symptoms (years)	Other causes of polyneuropathy	IS treatment	Follow-up (months)	NIS baseline	Neurological deterioration
Patient 1 (M)	Recurrence of HCV after first LT (HCV)	15	DM on insulin therapy,HbA1c:6.6%	Mycophenolate,1,000 mg/24 h	12	8	No
Patient 2 (M)	HCV LC	10	Vitamin B12 deficiency (normal B12 levels)	Mycophenolate,2,000 mg/24 h	64	8	Yes (12 months FU)
Patient 3 (F)	Alcoholic and HCV LC	7	DM on insulin therapy,HbA1c: 7.6–7.9%	Everolimus,1 mg/24 h	12	4	Yes (12 months FU)
Patient 4 (M)	HCV LC	9	—	Mycophenolate,1,000 mg/24 h	12	2	Yes (12 months FU)
				Everolimus,1.5 mg/24 h			
Patient 5 (F)	Alcoholic LC	7	DM on insulin therapy,HbA1c: <6%	Mycophenolic acid 1,080 mg/24 h	12	12	Yes (12 months FU)
Patient 6 (F)	HCV LC	13	—	Mycophenolate,1,000 mg/24 h	12	14	Yes (12 months FU)
Patient 7 (M)	HCV LC	13	—	Mycophenolic acid 1080mg/24 h	36	14	Yes (36 months FU)
Patient 8 (M)	Ischemic cholangitis after first LT (HCV LC)	9	—	Everolimus,1.5 mg/24 h	—	3	—
Patient 9 (M)	Thrombosis and rejection following LT (alcoholic and HBV LC)	6	DM on insulin therapy,HbA1c: 6.3%–6.6%	Mycophenolate,1,000 mg/24 h	—	0	—
				Everolimus,2 mg/24 h			
Patient 10 (M)	Chronic rejection after LT (HBV LC)	5	DM on insulin therapy,HbA1c:6%–6.1%	Tacrolimus 1 mg/24 h	--	6	--
				Azathioprine 100 mg/24 h			
Patient 11 (F)	Chronic rejection after LT	12	—	Tacrolimus 1 mg/24 h	—	12	—
Patient 12 (M)	HBV LC	11	DM on insulin therapy,HbA1c: 5.6%	Tacrolimus 0.5 mg/48 h	—	—	—

DLT, domino liver transplant; DM, diabetes mellitus; F, female; FU, follow-up; HbA1c, Glycated hemoglobin; HBV, hepatitis B virus; HCV, hepatitis C virus; IS, Immunosuppressive therapy; LC, liver cirrhosis; LT, liver transplant; M, male.

All liver donors had V30M genotypes and early-onset hATTR with neuropathic phenotypes. The mean time between transplant and symptom onset was 8.5 years (range, 5–15 years) (Tables 1, 2). The first manifestations of polyneuropathy were sensory, including painful dysesthesias and numbness in the feet (Table 3).

**TABLE 3:** Clinical findings at diagnosis of acquired amyloid neuropathy.

Patient	Weakness	Sensibility Disturbance	Autonomic Symptoms (*)	Neurological Examination
1	No	Dysesthesia and numbness in distal LL	No	Hypoesthesia in distal LL
2	No	Numbness in distal LL	Asthenia and weight loss	Tactile and thermal hypoesthesia in distal LL
				Tactile hypoesthesia in distal UL Absent Achilles reflex
3	No	Painful dysesthesia in distal LL	No	Hypopallesthesia in distal LL. Decreased Achilles reflex
4	No	Painful dysesthesia in distal LL	Erectile dysfunction	Thermal hypoesthesia in distal LL
5	No	Painful dysesthesia in distal LL	No	Thermo-algesic hypoesthesia and hypopallesthesia in distal LL
				Absent Achilles reflex
6	No	Painful dysesthesia in distal LL	Diarrhea	Tactile and thermo-algesic hypoesthesia in distal LL
				Hypopallesthesia in distal LL
				Absent patellar and Achilles’s reflex
7	No	Painful dysesthesia in distal LL	No	Tactile and thermo-algesic hypoesthesia in distal UL and LL
				Hypopallesthesia in distal LL
				Decreased Achilles reflex
8	No	Painful dysesthesia in distal LL	Erectile dysfunction, weight loss, diarrhea	Thermo-algesic hypoesthesia and hypopallesthesia in distal LL
9	No	Numbness in distal LL	Erectile dysfunction	Thermo-algesic hypoesthesia in distal UL and LL. Hypopallesthesia in distal LL
10	Yes	Painful dysesthesia in distal LL	No	Thermal hypoesthesia in distal LL
11	No	Painful dysesthesia in distal LL	Erectile dysfunction	Normal
12	Yes	Dysesthesia and numbness in UL and LL	Orthostatism, diarrhea	Thermo-algesic hypoesthesia and hypopallesthesia in UL and LL. Distal weakness in UL and LL.
				Absent patellar and Achilles’s reflex

(*) Excludes erectile dysfunction prior to domino liver transplant.

LL, lower limb; UL, upper limbs.

The median pretreatment scores were 10.8 (range, 0–46.5) for the NIS and 9.3 (range, 0–34.5) for the NIS-LL. All patients were Stage I–II of the Polyneuropathy Disability stage (PND) scale before starting diflunisal. Initial conventional neurophysiological study was normal in 2 patients (16.7%), but all patients developed a sensory-motor axonal polyneuropathy during the disease course. AAN was confirmed in all patients by the presence of amyloid deposition on sural nerve biopsy.

### Tolerability and Adverse Effects of Diflunisal

Diflunisal was started for compassionate use in all cases at a dosage of 250 mg twice daily as a stabilizing treatment. One patient received treatment for <6 months because he underwent re-transplantation. Among the remaining patients, five (45.5%) stopped treatment due to side effects (Table 4). Seven patients did persist with diflunisal for >12 months, but two of these (28.6%) required a dose reduction due to worsening renal function and one (14.3%) required that the drug be stopped due to heart failure. Two patients (28.6%) developed new-onset or worsening hypertension (Table 4), which was managed by adjusting antihypertensive therapy in all cases. All patients who developed impaired renal function showed a mild improvement in glomerular filtration rate after dose adjustment or stopping diflunisal, but none recovered to baseline levels.

**TABLE 4:** Diflunisal-related complications and dose changes.

	Renal function Worsening	Worsening or *de novo* AH	Discontinuation or Dose Reduction of diflunisal	Adverse Events after Therapy Modification
Patient 1 (M)	Yes (-12 ml/min, + 4 months)	No	Dose reduction to 250 mg/24 h due to renal function impairment (+5 months)	Mild improvement in renal function after dose reduction
Patient 2 (M)	Yes (-10 ml/min, +36 months)	Yes	Dose reduction to 250 mg/24 h (+59 months) due to renal function impairment	Mild improvement in renal function after dose reduction
			Discontinued due to heart failure (+64 months)	Heart failure recovery after discontinuation
Patient 3 (F)	No	No	No	—
Patient 4 (M)	No	No	No	—
Patient 5 (F)	No	No	No	—
Patient 6 (F)	No	No	No	—
Patient 7 (M)	No	No	No	—
Patient 8 (M)	Yes, acute renal failure in patient with chronic renal failure (EGFR<30 ml/min)	Yes	Discontinued due to acute renal failure (+13 days)	Mild improvement in renal function after discontinuation
Patient 9 (M)	—	—	No follow –up	—
			Liver re-transplantation	
Patient 10 (M)	—	—	Discontinued after acute cholestasis (+3 days)	—
Patient 11 (F)	—	—	Discontinued due to high hemorrhagic risk following anticoagulant therapy	—
Patient 12 (M)	Yes, acute renal failure in patient with chronic renal failure (EGFR<30 ml/min)	No	Discontinued due to acute renal failure (+35 days)	Mild improvement in renal function after discontinuation

AH, arterial hypertension; EGFR, Estimated Glomerular Filtration Rate; F, female; M, male.

### Treatment Efficacy

**FIGURE 1:**
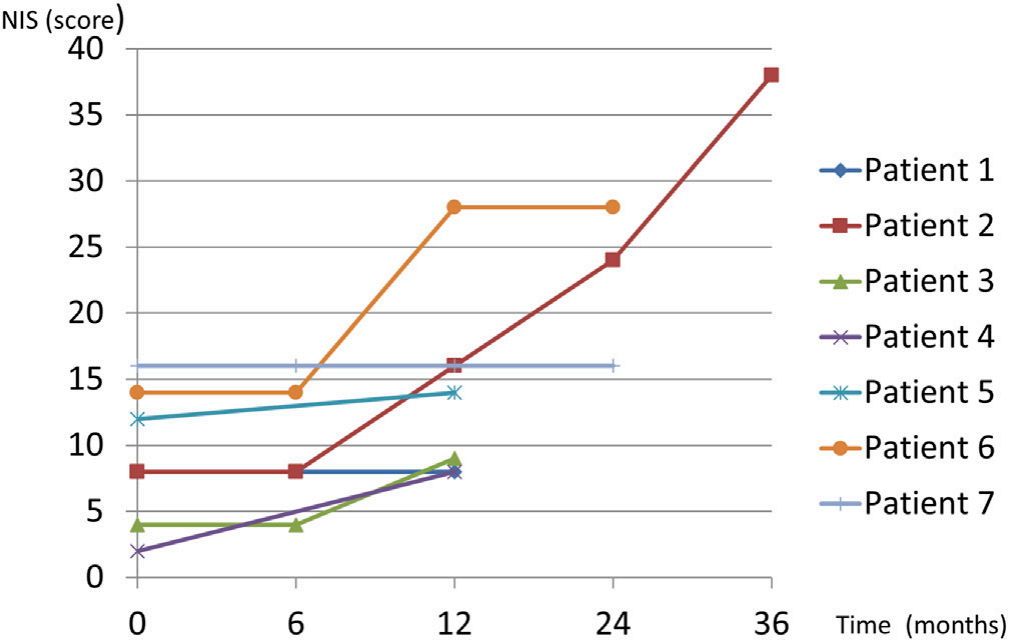
Diflunisal treatment in domino liver transplant recipients with acquired amyloid neuropathy.

Neurological follow-up data for at least 12 months after starting diflunisal were available for seven patients. The mean follow-up duration was 22.8 months (range, 12–36). No patient with assessment data at 6 months (4 patients, 57%) experienced neurological deterioration based on the NIS and NIS-L (Figure 1). However, five patients (71.4%) met the criteria for neurological deterioration at 12 months (Figure 1). Changes in the NIS from before treatment to 12 months of follow-up were statistically significant (*p* = 0.0382) whereas those in the NIS-LL were not (*p* = 0.09).

## Discussion

In the series presented by Misumi et al., the prevalence of symptomatic AAN among DLT recipients was 23% [20], whereas in our center, Lladó et al. reported that all patients had developed AAN at 90 months of follow-up [19]. Although liver re-transplantation is a viable treatment option, most patients are ineligible due to age or comorbidities [24, 28, 29], necessitating that we consider other treatment options. The generic nonsteroidal anti-inflammatory drug diflunisal is a nonspecific tetramer stabilizer of TTR that may prevent misfolding monomers and dimers from forming amyloid deposits in the heart and peripheral nerves [30].

A clinical trial has shown positive results on neurological progression when giving diflunisal to patients with hATTR [31], but evidence of its efficacy in DLT recipients with AAN is scarce. To date, there has only been one reported case of a patient with these features, which showed that neurological symptoms improved after 18 months of treatment [25]. Another patient who was given a trial of diflunisal needed their treatment to be stopped because of worsening heart failure [26]. Compassionate treatment with tafamidis was initiated in another patient with AAN, who remained stable for 2 years [27]. Prophylactic use of diflunisal or tafamidis has also been proposed in DLT recipients [32].

The efficacy of diflunisal in cardiac amyloidosis due to mutant or wild-type TTR has been analyzed in several studies [30, 33]. In our study we found that no patient had evidence of cardiac amyloidosis on cardiac scintigraphy before or during treatment in this case series. Only one patient developed heart failure at 36 months treatment, but this was without demonstrating cardiac amyloidosis (Supplementary material).

Our study is the first to analyze the effects of diflunisal in a series of seven patients with at least 12 months’ follow-up data. Before starting treatment, all patients were in stage I–II on the PND scale and stage I on Coutinho’s FAP scale. Most patients (71.4%) showed neurological deterioration by 1 year while only 28.6% remained stable on the NIS and NIS-LL. These results are similar to those reported in the clinical trial by Berk et al., in which 29.7% of patients with hATTR presented neurological stability (increase <2 points on the NIS +7 scale) after 2 years of treatment with diflunisal versus only 9.4% in the placebo group. It may be that a subgroup of patients responds to treatment and remains stable during the first years of treatment, as Bourque et al. [25] and Matsushima et al. [27] described. Analyzing the predictive factors of long-term response to diflunisal may be of benefit. Although we found that the change in NIS score at 12 months was statistically significant, whereas that for the NIS-LL score was not, it should be noted that these scales do not account for the proximal progression of sensory deficits and may underestimate deterioration.

In addition to the low efficacy we found high percentages of renal function impairment (36%), heart failure (9%), and treatment discontinuation (45%) in the medium/long-term course in our series that contrast with data in other studies of diflunisal for patients with hATTR or wild-type amyloidosis in which less renal function impairment was reported and diflunisal discontinuation occurred less often (0%–13%) [31, 30, 34]. This may be because DLT recipients are frail and have underlying comorbidities, with adverse effects being not only more common but also more likely to require drug cessation. Special attention must also be ensured for patients with chronic renal failure, poorly controlled hypertension, or receiving anticoagulants, ensuring close follow-up for possible complications. This data encourage the search for new therapeutic options.

Whether other treatment options for FAP, such as tafamidis [5], patisiran [6], or inotersen [7, 8], could be used with similar or better efficacy and fewer side effects in DLT recipients with AAN remains to be evaluated in prospective clinical trials.

### Limitations

The present series was limited by its retrospective nature, lack of a control group, small sample size, and inability to include follow-up data beyond 1 year for all patients. Nevertheless, sample size is an inherent problem of diseases with a low prevalence and a low rate of treatment continuation (54.5%).

Follow-up assessment of DLT recipients may be improved by using more sensitive scales such as the modified NIS + 7 together with a full neurological examination and the inclusion of functional scales.

## Conclusion

Our study suggests most of DLT recipients with AAN develop neurological deterioration after 12 months diflunisal treatment, and throughout, the high incidence of adverse effects frequently necessitates the drug being stopped. The low efficacy and the unfavorable side effect profile of diflunisal indicate that we need to identify new therapeutic options for patients who develop AAN after DLT.

## Data Availability

The original contributions presented in the study are included in the article/Supplementary Material, further inquiries can be directed to the corresponding author.

## Abbreviations

hTTRA, hereditary transthyretin-mediated amyloidosis; V30M, Val30Met; TTR, transthyretin; DLT, domino liver transplantation; FAP, familial amyloid neuropathy; AAN, acquired amyloid neuropathy; UMAF, Familial Amyloidosis Multidisciplinary Unit; NIS, Neurological Impairment Scale; NIS-LL, Neurological Impairment Scale-Lower Limbs; PND, Polyneuropathy Disability score; HCV, hepatitis C virus; HBV, hepatitis B virus; LC, liver cirrhosis; DLT, domino liver transplant; LT, liver transplant; DM, diabetes mellitus; M, male; UL, upper limbs; LL, lower limb.
