# Supplementary material for: Treatment With Diflunisal in Domino Liver Transplant Recipients With Acquired Amyloid Neuropathy
Source: Transpl Int. 2022 Apr 13;35:10454. doi: 10.3389/ti.2022.10454 (PMC9044119; doi:10.3389/ti.2022.10454)
Supplement: Supplementary file 1 [file Table1.docx]

**Supplementary material.** Cardiac assessment before and during treatment with diflunisal

|  | NYHA  0 year | NYHA 1 year | BNP  Initial | BNP  1 year | BNP 2 year | Scintigraphy  Pretreatment | Scintigraphy at follow-up | Echocardiogram  Pretreatment | Echocardiogram  1 year |
| --- | --- | --- | --- | --- | --- | --- | --- | --- | --- |
| Patient  1 | **1** | **1** | **1760** | **1871** | **1197** | **Score 0** | **Score 0** | **Mild LV hypertrophy. Normal EF. Repaired mitral valve.** | **No changes.** |
| Patient  2 | **2** | **2** | **114** | **432** | **447** | **Score 0** | **--** | **Mild LV hypertrophy. Normal EF.**  **Mild mitral regurgitation.** | **No changes.** |
| Patient  3 | **1** | **1** | **85** | **124** | **126** | **Score 0** | **Score 0** | **Mild septal hypertrophy. Normal EF.** | **No changes.** |
| Patient  4 | **1** | **1** | **110** | **146** | **125** | **Score 0** | **Score 0** | **Mild septal hypertrophy. Normal EF.** | **No changes.** |
| Patient  5 | **1** | **1** | **507** | **--** | **740** | **Score 0** | **Score 0** | **Normal.** | **..** |
| Patient  6 | **1** | **1** | **312** | **--** | **512** | **Score 0** | **--** | **Normal.** | **No changes.** |
| Patient  7 | **1** | **1** | **537** | **452** | **460** | **Score 0** | **Score 0** | **Mild LV hypertrophy**  **Mild LA enlargement** | **No changes.** |
| Patient  8 | 1 | 1 | 712 | -- |  | Score 0 | -- | Mild LV hypertrophy | -- |
| Patient  9 | 1 | 1 | 205 | 100 |  | Score 0 | Score 0 | Mild septal hypertrophy | No changes. |
| Patient 10 | 1 | 1 | 491 | 580 |  | Score 0 | Score 0 | Mild LV hypertrophy | No changes. |
| Patient 11 | 2 | 2 | 2040 | 1648 | 1294 | Score 0 | Score 0 | Mild LV hypertrophy. LA and RA enlargement | No changes. |
| Patient 12 | 1 | 1 | -- | -- | -- | -- | -- | Mild LV hypertrophy | -- |

Perugini grading scale in cardiac scintigraphy with 99mTc-DPD (grade 0: no cardiac uptake and normal bone uptake; grade 1: cardiac uptake which is less than bone uptake; grade 2: cardiac uptake with intensity similar to or greater than bone uptake; grade 3: cardiac uptake with much reduced or absent bone signal).

BNP: NT-proBNP (ng/L, normal < 300 ng/L). EF: ejection fraction. LA: left atrium. LV: left ventricle.
